# The relationship between risk perception, anxiety and paranoia – A predictive model in a community sample

**DOI:** 10.1016/j.xjmad.2024.100052

**Published:** 2024-01-20

**Authors:** Suzanne H. So, Anson Kai Chun Chau, Brandon A. Gaudiano, Lyn Ellett, Tania M. Lincoln, Eric M.J. Morris, Jessica L. Kingston

**Affiliations:** a Department of Psychology, The Chinese University of Hong Kong, Hong Kong Special Administrative Region; b Institute of Health Equity, The Chinese University of Hong Kong, Hong Kong Special Administrative Region; c Department of Psychiatry and Human Behavior, Brown University, Providence, RI, USA; d School of Psychology, University of Southampton, Southampton, UK; e Department of Clinical Psychology and Psychotherapy, University of Hamburg, Hamburg, Germany; f School of Psychology and Public Health, La Trobe University, Melbourne, Australia; g Department of Psychology, Royal Holloway, University of London, London, UK

**Keywords:** Persecutory, Paranoid ideation, Psychosis, Worry, Transdiagnostic

## Abstract

**Background:**

Biases in risk perception (e.g. excessive attribution of likelihood of negative events happening to oneself, or perceived harm of neutral events) have been suggested as risk factors for psychopathologies such as generalised anxiety and persecutory ideation, although this line of research is limited by small samples and a lack of a suitable risk perception scale.

**Methods:**

Using the Risk Perception Questionnaire, four risk perception dimensions (likelihood, harm, controllability, and intentionality) of negative and neutral events were tested in association with anxiety and paranoia. In view of common co-occurrence between the two symptom variables, their associations with risk perception were tested by using partial correlations (at baseline) and comparisons of cross-lagged path models (over 3 months).

**Results:**

A representative community-based sample of 445 adults were included. At baseline, after controlling for correlations between levels of anxiety and paranoia, anxiety was uniquely correlated with three risk perception dimensions for negative events (likelihood, harm, and intentionality), whereas paranoia was uniquely correlated with all risk perception dimensions for both negative and neutral events. The best-fitted cross-lagged path model revealed that, after controlling for auto-regressions within variables, baseline level of anxiety predicted perceived harm of negative events at 3 months, whereas baseline levels of perceived intentionality of neutral events and likelihood of negative events predicted level of paranoia at 3 months.

**Conclusions:**

While risk perception of negative events is shared between anxiety and paranoia, risk perception of neutral events is uniquely characteristic of paranoia. Implications on maintenance of sub-clinical symptoms are discussed.

## Highlights

• Risk perception consists of likelihood, harm, controllability, and intentionality.
• Risk perception of negative events is shared between anxiety and paranoia.
• Risk perception of neutral events is uniquely characteristic of paranoia.

## 1 Introduction

Risk perception has traditionally been defined as the subjective assessment of the probability of an adverse event and the seriousness of its consequences [40], [44]. The concept has widely been applied to research in natural hazards (e.g. [41]; [42], pandemic outbreaks (e.g. [43]; [54], and preventable accidents such as drunk driving (e.g. [53], with a focus on increasing public awareness and ability to manage disasters.

On the other hand, excess and biases in risk perception have been observed in individuals with various psychopathologies. For example, individuals with paranoia tend to anticipate threat to themselves even without objective evidence [15], [16], [12]. Compared to non-anxious individuals, those with general anxiety also tend to perceive negative events as more likely to happen to them and to cause more harmful consequences [3], [6], [30]. Apart from biases in perceived likelihood and harm, a lower level of perceived controllability has also been reported in individuals with paranoia [33], [39] and general anxiety [49], [48] respectively. Lastly, attributing intentionality to adverse events has been suggested as a core feature of paranoia, which may be distinct from general anxiety [11]. For individuals with anxiety and paranoia, biases in risk perception are linked to distress and engagement in ‘safety behaviours’, which only lead to short-term relief but maintain their symptoms in the longer term [1], [14], [18], [35].

While biased and excessive perception of risks may be characteristic of general anxiety and paranoia, most studies in this area focused on one of the two symptoms only. Given that anxiety and paranoia commonly co-occur in clinical and sub-clinical populations (e.g. [36]; [51], and that anxiety may be a precedent of paranoia [23], [24], [25], it would be of interest to evaluate how subclinical levels of paranoia can be differentiated from anxiety in the general population, and how these symptoms relate to risk perception within the same sample. In addition, as opposed to traditional risk perception research that focused on adverse events and dangers only, understanding of risk perception biases in psychopathologies would benefit from a comprehensive assessment of both negative and neutral events where over-sensitivity to risks may be revealed.

In order to provide a comprehensive assessment of risk perception for studying psychopathologies such as anxiety and paranoia, Chan et al. [7] developed a Risk Perception Questionnaire (RPQ). As an extension of the work of Kaney et al. [20] and Freeman et al. [16], the RPQ consists of 10 hypothetical neutral events and 15 hypothetical negative events, both including social scenarios. While other risk perception tools include likelihood and harm/consequences only, the RPQ encompasses four dimensions of subjective risk evaluation: (i) likelihood of the event happening to them in the near future, (ii) harm of the event, (iii) controllability of the event and its consequences, and (iv) intentionality of someone for this event to happen. Controllability and intentionality were added in response to suggestions by risk perception researchers [31], [32], [45], [5] and clinical observations, e.g. the tendency for individuals with paranoia to attribute negative events to personal intentions [2], [38].

Using the RPQ, So et al. [47] compared risk perception dimensions across groups of individuals with anxiety and paranoia for the first time. The first part of that study consisted of a comparison between patients with psychotic disorders experiencing persecutory delusions, patients with generalized anxiety disorder, and healthy controls. The second part was between non-clinical individuals with paranoia, individuals with general anxiety, and healthy controls. They found that, in both parts of the study, perceived likelihood of negative events was heightened in the two symptomatic groups as opposed to healthy controls, whereas perceived harm of neutral events was uniquely heightened in groups with paranoia.

Uncovering the similarities and differences across psychopathologies may provide insights for designing intervention that targets these nuances across clinical groups. However, So et al. [47] was limited by a small sample size (each group including 21–52 participants) and a cross-sectional design. The non-clinical arm included university students only, limiting sample representativeness. More importantly, individuals were categorized into groups based on clinical assessment (in the first comparison) and cluster analysis (in the second comparison), yielding arbitrary groups that are at certain points of the symptom spectrum only.

The present study aimed to extend our understanding on the relationship between risk perception dimensions, anxiety and paranoia in a representative and sizable community-based sample that spans across the full spectrum of the adult population. As sub-clinical symptoms can change or impact on each other over time (e.g. [52], the present study also aimed to examine the directionality of associations between risk perception dimensions and the two symptoms using a repeated-measures design (with two timepoints three months apart). Therefore, baseline relationships between the key variables were first examined using partial correlation analyses, whereas relationships across time were evaluated by cross-lagged path analyses using a model comparison approach. This investigation will provide insights into risk perception as a contributor to the two often co-occurring symptoms (e.g. [4]; [15], and hence a potential target for intervention.

Major hypotheses are as follows.

1. At baseline, perceived likelihood of negative events will be positively associated with levels of anxiety and paranoia, whereas perceived controllability of negative events will be negatively associated with levels of anxiety and paranoia.
2. At baseline, perceived harm of neutral events and perceived intentionality of negative events will be more positively associated with level of paranoia than that of anxiety.
3. Perceived likelihood of negative events at baseline will predict subsequent increases in anxiety and paranoia, whereas perceived controllability of negative events will predict subsequent decreases in anxiety and paranoia.
4. Perceived harm of neutral events and perceived intentionality of negative events at baseline will predict subsequent increases in paranoia.

## 2 Methods

Ethical approval was obtained from the university’s Survey and Behavioural Research Ethics Committee (ref. no. SBRE-20–233). Written consent was obtained from all participants.

### 2.1 Participants

Inclusion criteria were as follows: adults age 18 or above; ability to fill out an online survey in Chinese; currently living in Hong Kong. No exclusion criteria were applied. The sample was recruited via the Qualtrics panel service using stratified quota sampling based on sex, age, and educational attainment. While the sample was part of an international consortium on paranoia [10], [22], [26], [46], the analysis in this paper is new and has not been reported elsewhere.

Using the R-package semTools [19], a sample size calculation yielded a minimum sample size of 228 for the most complex path analytical model of 20 variables (RMSEA = 0.05, power = 0.80, alpha = 0.05, df = 56). After taking into account 20% potential dropouts, a sample size of 285 would be needed at baseline.

### 2.2 Measures

#### 2.2.1 Risk perception

Risk perception was assessed with the Risk Perception Questionnaire (RPQ; [47], [7], which consists of 15 negative scenarios and 10 neutral scenarios. For each scenario, participants are required to rate their perceived likelihood of happening in the near future, perceived harm, perceived controllability and perceived intentionality on 7-point Likert scales (1 = ‘Not at all’, 7 = ‘Very much’). Four average dimensional scores for negative and neutral scenarios are reported separately (range = 1 to 7). Internal reliabilities of the four subscales for both event types were good in the current sample (Cronbach’s αs at baseline: 0.88–0.94 (neutral events) and 0.92–0.93 (negative events); Cronbach’s αs at follow-up: 0.92–0.93 (neutral events) and 0.92–0.94 (negative events)). See Appendix 1 for the RPQ scale items.

#### 2.2.2 Mood variables

The Depression, Anxiety and Stress Scale – 21 Items (DASS-21, [29]) was used to assess depression, anxiety, and stress. Each subscale consists of seven items, with a subscale score ranging from 0 to 21. Good psychometric properties of the Chinese version are reported (e.g. [34]). The internal consistencies were good in the current sample (Cronbach’s αs at baseline: 0.91 (Depression), 0.87 (Anxiety), and 0.89 (Stress); Cronbach’s αs at follow-up: 0.90 (Depression), 0.84 (Anxiety), and 0.90 (Stress)).

#### 2.2.3 Paranoia

Paranoia was assessed with the persecution subscale of the Revised Green et al. Paranoid Thoughts Scale (R-GPTS; [17]. The persecution subscale of the R-GPTS is a 10-item 5-point (0 to 4) rating scale assessing ideas of persecution (ten items) in the general population with established cut-off scores (Average: 0–5; Elevated: 6–10; Moderately severe: 11–17; Severe: 18–27; Very severe: 28 +). The Chinese version of the R-GPTS has been used in previous studies (e.g. [46], [8]). The persecution subscale of the R-GPTS had excellent internal consistency in the current sample (Cronbach’s α: 0.96 at baseline and 0.95 at follow-up).

#### 2.2.4 Other measures

Participants also provided information on age, gender, household income, and current diagnosis of a mental health disorder (“Have you been diagnosed with any psychiatric disorder?” – Yes/no) at baseline.

### 2.3 Procedure

Upon written consent, participants were asked to fill out an online survey on the Qualtrics platform. Three months later, they were invited to fill out the same set of questionnaires. Forced responses were set in each survey, preventing missing data. A careful validity check procedure was adopted: machine responses, duplicates, and responses that were completed too quickly (less than half of the median completion time) or inattentively (failing the five attention check items) were removed [46]. Only validated responses were entered into data analysis.

## 3 Calculation

To test Hypotheses 1–2, the unique relationship between a symptom variable (e.g. anxiety) and each risk perception dimension was examined using partial Spearman’s rank correlation analysis, adjusting for the relationship with the other symptom variable (e.g. paranoia). These analyses were conducted on JAMOVI.

To examine the longitudinal relationships between symptom variables and risk perception dimensions (Hypotheses 3–4), a series of path models were tested by using the R package lavaan. First, we regressed all variables at three months on their values at baseline (i.e. autoregressive paths) (Model 1). The next models added cross-lagged paths across variables on top of the autoregressive model in a step-by-step manner. Specifically, Model 2a included autoregressive paths *and* the cross-lagged paths between the two symptom variables, which have been hypothesized in previous studies (e.g. [52]. Model 2b included autoregressive paths *and* the cross-lagged paths from risk perception dimensions to symptoms. Model 2c included the autoregressive paths *and* the cross-lagged paths from symptoms to risk perception dimensions. Finally, Model 3 incorporated significant cross-lagged path(s) from the above models. The absolute fit of the models was evaluated by the Comparative Fit Index (CFI), Tucker-Lewis Index (TLI), Root Mean Squared Error of Approximation (RMSEA) and Standardized Root Mean Squared Residual (SRMR). According to recommendations in Hu and Bentler (1998) and Marsh et al. (2004), CFI and TLI values > 0.90 are acceptable and > 0.95 are excellent. RMSEA and SRMSR values < 0.10 are acceptable and < 0.05 are excellent. Goodness-of-fit was compared across models on likelihood ratio tests, where a significant test statistic would indicate preference for the more complex model over the less complex one. Attrition was handled with listwise deletion.

As depression may be associated with both anxiety and paranoia, to test for result robustness, post-doc analyses controlling for depression (measured by DASS-D) were conducted for both cross-sectional and longitudinal analyses.

## 4 Results

### 4.1 Sample characteristics

A total of 524 individuals responded to the online survey. Among them, 445 (84.9%) passed the validity check and were entered into the baseline analysis. At 3-month follow-up, a total of 296 participants completed the survey and passed the validity check, resulting in a retention rate of 66.5%.

The baseline sample (N = 445) had a mean age of 39.64 (SD = 13.58), with 252 (56.6%) females. Detailed demographic characteristics of the sample can be found in Kingston et al. [22]. The great majority (92.8%) of the sample did not have a self-reported mental health diagnosis. The mean DASS scores were as follows: DASS-total (14.78, SD = 12.77), DASS-D (4.62, SD = 4.66), DASS-A (4.34, SD = 4.24), DASS-S (5.81, SD = 4.69). The mean R-GPTS Persecution subscore was 8.52 (SD = 9.67). The sample distribution on the R-GPTS Persecution subscore was as follows: ‘average’ = 55.1%; ‘elevated’ = 11.7%; ‘moderately severe’ = 11.7%; ‘severe’ = 16.2%; ‘very severe’ = 5.4% [17].

### 4.2 Relationships between risk perception dimensions, anxiety and paranoia at baseline

Partial correlations between risk perception dimensions, anxiety and paranoia, are shown in Table 1. Correlations between major variables and demographic characteristics are shown in Appendix 2.

**Table 1:** Partial correlations between risk perception and symptoms at baseline (N = 455).

Variables	DASS-A (controlling for R-GPTS Persecution)		R-GPTS Persecution (controlling for DASS-A)	
	*ρ*	*p*	*ρ*	*p*
RPQ-neutral: likelihood	-0.02	.712	0.18	< .001
RPQ-neutral: harm	0.09	.059	0.33	< .001
RPQ-neutral: controllability	-0.01	.855	0.16	< .001
RPQ-neutral: intentionality	-0.04	.447	0.24	< .001
RPQ-negative: likelihood	0.24	< .001	0.30	< .001
RPQ-negative: harm	0.30	< .001	0.28	< .001
RPQ-negative: controllability	0.03	.485	0.24	< .001
RPQ-negative: intentionality	0.11	.025	0.31	< .001

After controlling for R-GPTS Persecution subscore, the DASS-A score was significantly and positively correlated with three risk perception dimensions for *negative* events (likelihood, harm, and intentionality [*ρs* = 0.11–0.30, *ps* <.050]). After controlling for R-GPTS Persecution subscore, the DASS-D score was also significantly and positively correlated with these three dimensions of *negative* events (*ρs* = 0.14–0.30, *ps* <.050), as well as perceived harm of *neutral* events (*ρ* = 0.09, *p* = .049). Post-hoc analysis revealed that, after controlling for R-GPTS Persecution subscore and the DASS-D score, the DASS-A score remained significantly and positively correlated with perceived likelihood and harm of *negative* events (*ps* <.050).

After controlling for DASS-A, the R-GPTS Persecution subscore was significantly and positively correlated with all risk perception dimensions for both *negative* and *neutral* events (*ρs* = 0.16–0.33, *ps* <.001). Post-hoc analysis revealed that, after controlling for DASS-A and DASS-D, the R-GPTS Persecution subscore remained significantly and positively correlated with all risk perception dimensions for both *negative* and *neutral* events (*ps* <.001).

The above findings were not moderated by age. When male and female participants were analysed separately, most findings remained unchanged except for the partial associations of R-GPTS Persecution subscore with perceived controllability in both neutral and negative events, which were significant in females but not in males.

### 4.3 Relationships between risk perception dimensions, anxiety and paranoia over 3 months

As shown in Table 2, there were significant reductions in anxiety, stress and paranoia over three months (*ps* <.050). There were significant reductions in perceived likelihood and controllability for both *negative* and *neutral* events (*ps* <.050), as well as perceived intentionality for *neutral* events only. Perceived harm for both *negative* and *neutral* events did not change significantly over time (*ps* >.050).

**Table 2:** Major variables at each time point.

	Baseline (N = 455)	Follow-up (N = 296)	Follow-up vs. baseline
DASS-D	4.62 (4.66)	3.72 (0.24)	W = 12,906.50, p = .069
DASS-A	4.34 (4.24)	3.23 (3.49)	W = 15,268.50, *p* < .001
DASS-S	5.81 (4.69)	4.96 (4.35)	W = 17,915.00, p = .020
R-GPTS Persecution	8.52 (9.67)	7.02 (8.24)	W = 15,126.00, *p* = .027
RPQ-neutral: likelihood	3.90 (1.27)	3.75 (1.24)	W = 21,821.50, *p* = .006
RPQ-neutral: harm	1.79 (1.04)	1.59 (0.84)	W = 13,688.50, *p* = .105
RPQ-neutral: controllability	3.33 (1.47)	3.16 (1.42)	W = 22,382.00, *p* < .001
RPQ-neutral: intentionality	3.59 (1.52)	3.35 (1.51)	W = 25,252.50, *p* < .001
RPQ-negative: likelihood	3.17 (1.15)	2.98 (1.08)	W = 23,370.50, *p* = .032
RPQ-negative: harm	3.50 (1.46)	3.42 (1.46)	W = 21,809.50, *p* = .357
RPQ-negative: controllability	3.06 (1.28)	2.87 (1.91)	W = 23,142.00, *p* = .020
RPQ-negative: intentionality	3.60 (1.37)	3.41 (1.37)	W = 22,416.00, *p* = .072

Note: DASS = Subscales of the Depression Anxiety Stress Scale (D = Depression; A = Anxiety; S = Stress). R-GPTS = Revised Green Paranoid Thoughts Scale. RPQ = Risk Perception Questionnaire.

**Fig. 1:**
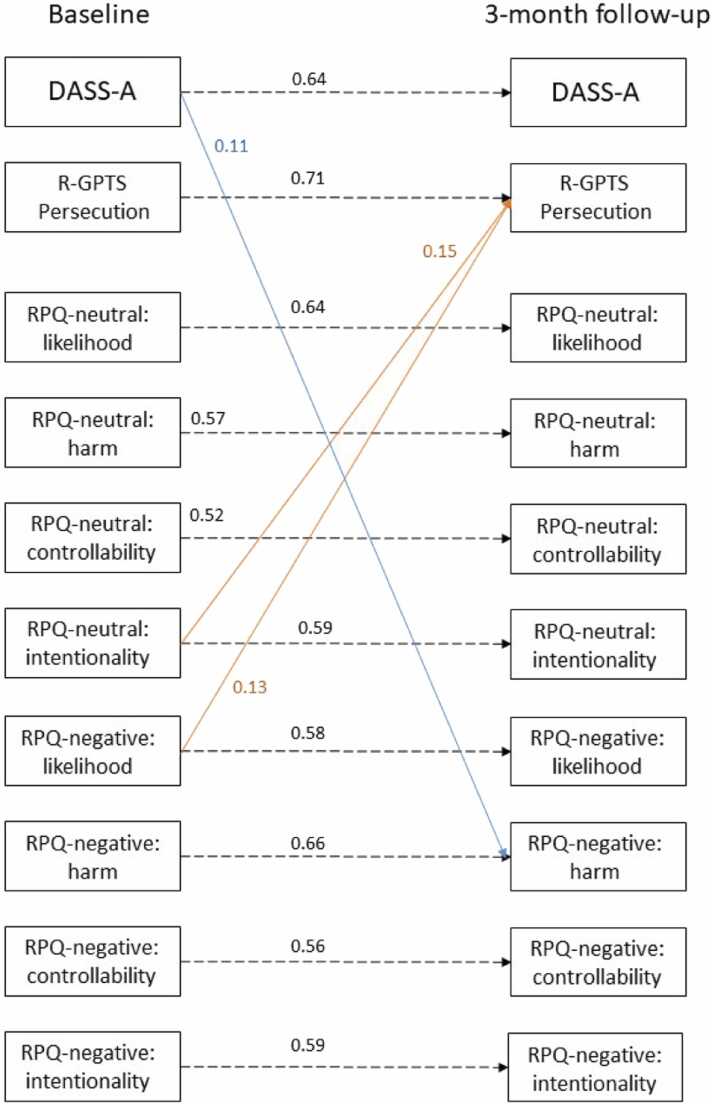
The final path model (Model 3) of the relationships between anxiety, paranoia and risk perception across time points. Note: Only significant paths are shown. Autoregressive paths are indicted by dotted lines, whereas cross-lagged paths are indicated by solid lines. Values given are standardized coefficients. DASS = Subscales of the Depression Anxiety Stress Scale (D = Depression; A = Anxiety; S = Stress). R-GPTS = Revised Green Paranoid Thoughts Scale. RPQ = Risk Perception Questionnaire.

Various longitudinal path analysis models and their goodness-of-fit indexes are listed in Table 3. The best-fitted model was Model 3 (see Fig. 1). In addition to auto-regressions within variables, baseline level of anxiety significantly predicted level of perceived harm of *negative* events at three months. Baseline levels of perceived intentionality of *neutral* events and likelihood of *negative* events predicted level of paranoia at three months. These cross-lagged paths were not moderated by age or gender.

**Table 3:** Goodness-of-fit indexes of various longitudinal path analyses.

Models	χ^2^ (df)	CFI	TLI	RMSEA	SRMR	Likelihood ratio test (χ^2^ (df))
Model 1: All auto-regressive paths	138.10(90), *p* = .001	0.99	0.97	0.04	0.07	/
Model 2a: Model 1 plus cross-lagged paths between symptoms	133.01(88), *p* = .001	0.99	0.97	0.04	0.06	vs. Model 1: 4.74(2), *p* = .094
Model 2b: Model 1 plus cross-lagged paths from risk perception to symptoms	112.56(74), *p* = .003	0.99	0.97	0.04	0.06	vs. Model 1: 29.50(16), *p* = .020
Model 2c: Model 1 plus cross-lagged paths from symptoms to risk perception	107.83(74), *p* = .006	0.99	0.98	0.04	0.05	vs. Model 1: 50.32(16), *p* = .019
Model 3: Model 1 plus cross-lagged paths between risk perception and symptoms	83.36(58), *p* = .016	0.99	0.98	0.04	0.04	vs. Model 1: 58.72(32), *p* = .002 vs. Model 2a: 52.93(30), *p* = .006 vs. Model 2b: 29.22(16), *p* = .022 vs. Model 2c: 28.27(16), *p* = .029

As an exploratory analysis, in a separate model baseline level of depression predicted levels of perceived intentionality (β = 0.09, *p* = .034) and harm (β = 0.15, *p* = .001) of *negative* events. Baseline level of perceived likelihood of *negative* events marginally predicted level of depression at follow-up (β = 0.12, p = .058).

Post-hoc analyses revealed that, after controlling for levels of depression at baseline and follow up, the cross-lagged path from anxiety to perceived harm of *negative* events became non-significant (*p* > .050). However, the two cross-lagged paths from risk perception to paranoia remained statistically significant (*ps* <.050).

## 5 Discussion

This study investigated the relationship between risk perception dimensions and two typically co-occurring symptoms, anxiety and paranoia, in a sizable and representative community-based sample. By including anxiety and paranoia in the same model, and by re-assessing the variables over three months, this study offered a proof of concept for temporal relationships after controlling for auto-regressions. Although it has been argued that multi-dimensional assessment for risk perception may not be needed because dread and novelty of risks explain the most variance (review by [44], our findings lend support to the use of a multi-dimensional assessment, which allowed for tests of specific associations between symptoms and four dimensions of risk perception (likelihood, harm, controllability, and intentionality) towards negative and neutral events.

Cross-sectionally, anxiety was uniquely (and positively) correlated with three risk perception dimensions for negative events after controlling for correlations between the two symptoms. On the other hand, paranoia was uniquely (and positively) correlated with all four risk perception dimensions for *both* negative and neutral events. Even though the correlation coefficients were rather small, most of these correlations remained robust after controlling for level of depression. Consistent with So et al. [47], while the associations with risk perception for negative events were shared with anxiety, the associations with risk perception for neutral events were unique for paranoia. Clinical studies on anxiety and psychosis have suggested that some psychological processes may be transdiagnostically relevant, such as worry and safety behavior (e.g. [37]; [51], [52], supporting the idea that paranoia can be understood using anxiety models (e.g. [50]. Our findings add risk perception for negative events to the list of shared processes between the two symptoms.

Importantly, a sensitized perception towards neutral events appears to be characteristic of paranoia only. In particular, increased perceived intentionality of neutral events at baseline predicted an increase in paranoia at three months, even after controlling for paranoia and anxiety at baseline. Even though the regression coefficients were small, they remained robust after controlling for levels of depression. The tendency to misinterpret neutral events and social exchanges as harmful and intentional is consistent with cognitive models of psychosis and paranoia that highlight the roles of appraisal and reasoning biases, biases in facial affect recognition, and over-mentalising (e.g. [9]; [13]. On the contrary, the cross-sectional associations between perception of likelihood and controllability of neutral events and paranoia is less expected. While the increased perception of likelihood of neutral events may be understood in the context of aberrant salience (i.e. an anomalous sense of novelty and significance to irrelevant stimuli; Kapur [21], which is more evident in patients with persecutory delusions than sub-clinical samples, the increased perception of controllability of both neutral and negative events should be interpreted with caution.

The temporal relationship between the two symptoms and risk perception dimensions were formally tested for the first time to our knowledge, with the hypotheses that risk perception would predict increases in symptoms. This investigation is considered stringent because auto-regressions were controlled for, and cross-lagged paths between symptoms were tested before paths between symptoms and risk perception, in a step-by-step manner. We found that only change in paranoia was predicted by baseline risk perception (in particular, likelihood of negative events and intentionality of neutral events), which survived significance after controlling for depression; while anxiety at baseline predicted subsequent increase in risk perception (harm of negative events), this path became non-significant after controlling for depression.

Some non-findings were noted. For example, perceived controllability of negative events did not predict change in anxiety and paranoia, and perceived harm of neutral events and intentionality of negative events did not predict increases in paranoia. Until future studies investigating these temporal associations emerge, interpretation of these non-findings can only be speculative. Although it has been theorized that perception of future threat initiates the anxiety and paranoia processes, it has also been suggested that emotions (including anxiety) play a significant role in shaping risk perception [28], [27]. Therefore, risk perception may be a less stable construct than previously thought, interacting with symptoms in a dynamic way. Future research might usefully examine the dynamic interplay between risk perception and symptoms.

This study was limited by the fact that assessment of symptoms and risk perception was based on self-report. As argued by Loewenstein et al. [28], responses to risky situations (including decision making) may result in part from direct emotional influences. Therefore, it would have been better if affective aspects of risk were also measured. Since the RPQ measures negative and neutral events only, it is also unclear how risk perception is like for other emotionally-valenced events such as positive events. Secondly, despite efforts of retaining participants in the follow-up survey, the completion rate still fell short of 70%. Thirdly, it is unclear how findings based on this non-clinical sample would translate to a more clinically severe population.

To conclude, the present results revealed that while risk perception of negative events may be shared between anxiety and paranoia, risk perception of neutral events may be uniquely characteristic of paranoia. If these results are further confirmed by future studies, ideally across cultural groups, they would inform identification of specific intervention foci for anxiety and paranoia respectively in sub-clinical groups.

## Ethical standards

The authors assert that all procedures contributing to this work comply with the ethical standards of the relevant national and institutional committees on human experimentation and with the Helsinki Declaration of 1975, as revised in 2008.

## Declaration of Competing Interest

None.

## Figures and Tables

**Table:** 

Someone asks you to pass them the sugar	not at all			very much			
a. How likely will this event happen to you in the near future?	1	2	3	4	5	6	7
b. How intentional is this event?	1	2	3	4	5	6	7
c. How controllable is this event by you?	1	2	3	4	5	6	7
d. How harmful is this event to you?	1	2	3	4	5	6	7

**Table:** 

Someone reads your mail without your permission	not at all			very much			
a. How likely will this event happen to you in the near future?	1	2	3	4	5	6	7
b. How intentional is this event?	1	2	3	4	5	6	7
c. How controllable is this event by you?	1	2	3	4	5	6	7
d. How harmful is this event to you?	1	2	3	4	5	6	7

**Table:** 

You are handed a leaflet in the street	not at all			very much			
a. How likely will this event happen to you in the near future?	1	2	3	4	5	6	7
b. How intentional is this event?	1	2	3	4	5	6	7
c. How controllable is this event by you?	1	2	3	4	5	6	7
d. How harmful is this event to you?	1	2	3	4	5	6	7

**Table:** 

Someone stares at you menacingly	not at all			very much			
a. How likely will this event happen to you in the near future?	1	2	3	4	5	6	7
b. How intentional is this event?	1	2	3	4	5	6	7
c. How controllable is this event by you?	1	2	3	4	5	6	7
d. How harmful is this event to you?	1	2	3	4	5	6	7

**Table:** 

You are asked to sign a petition	not at all			very much			
a. How likely will this event happen to you in the near future?	1	2	3	4	5	6	7
b. How intentional is this event?	1	2	3	4	5	6	7
c. How controllable is this event by you?	1	2	3	4	5	6	7
d. How harmful is this event to you?	1	2	3	4	5	6	7

**Table:** 

You are followed down the road	not at all			very much			
a. How likely will this event happen to you in the near future?	1	2	3	4	5	6	7
b. How intentional is this event?	1	2	3	4	5	6	7
c. How controllable is this event by you?	1	2	3	4	5	6	7
d. How harmful is this event to you?	1	2	3	4	5	6	7

**Table:** 

You receive mail about a new offer	not at all			very much			
a. How likely will this event happen to you in the near future?	1	2	3	4	5	6	7
b. How intentional is this event?	1	2	3	4	5	6	7
c. How controllable is this event by you?	1	2	3	4	5	6	7
d. How harmful is this event to you?	1	2	3	4	5	6	7

**Table:** 

Someone tells you that you are stupid	not at all			very much			
a. How likely will this event happen to you in the near future?	1	2	3	4	5	6	7
b. How intentional is this event?	1	2	3	4	5	6	7
c. How controllable is this event by you?	1	2	3	4	5	6	7
d. How harmful is this event to you?	1	2	3	4	5	6	7

**Table:** 

Someone stops you to ask directions	not at all			very much			
a. How likely will this event happen to you in the near future?	1	2	3	4	5	6	7
b. How intentional is this event?	1	2	3	4	5	6	7
c. How controllable is this event by you?	1	2	3	4	5	6	7
d. How harmful is this event to you?	1	2	3	4	5	6	7

**Table:** 

You are told that you are ugly	not at all				very much		
a. How likely will this event happen to you in the near future?	1	2	3	4	5	6	7
b. How intentional is this event?	1	2	3	4	5	6	7
c. How controllable is this event by you?	1	2	3	4	5	6	7
d. How harmful is this event to you?	1	2	3	4	5	6	7

**Table:** 

A ticket collector tells you how much a fare is	not at all				very much		
a. How likely will this event happen to you in the near future?	1	2	3	4	5	6	7
b. How intentional is this event?	1	2	3	4	5	6	7
c. How controllable is this event by you?	1	2	3	4	5	6	7
d. How harmful is this event to you?	1	2	3	4	5	6	7

**Table:** 

Someone tells you that you are deceitful	not at all				very much		
a. How likely will this event happen to you in the near future?	1	2	3	4	5	6	7
b. How intentional is this event?	1	2	3	4	5	6	7
c. How controllable is this event by you?	1	2	3	4	5	6	7
d. How harmful is this event to you?	1	2	3	4	5	6	7

**Table:** 

Someone asks you if the seat next to you is taken	not at all				very much		
a. How likely will this event happen to you in the near future?	1	2	3	4	5	6	7
b. How intentional is this event?	1	2	3	4	5	6	7
c. How controllable is this event by you?	1	2	3	4	5	6	7
d. How harmful is this event to you?	1	2	3	4	5	6	7

**Table:** 

Someone complains about your work	not at all				very much		
a. How likely will this event happen to you in the near future?	1	2	3	4	5	6	7
b. How intentional is this event?	1	2	3	4	5	6	7
c. How controllable is this event by you?	1	2	3	4	5	6	7
d. How harmful is this event to you?	1	2	3	4	5	6	7

**Table:** 

Someone asks if they may borrow your pen.	not at all				very much		
a. How likely will this event happen to you in the near future?	1	2	3	4	5	6	7
b. How intentional is this event?	1	2	3	4	5	6	7
c. How controllable is this event by you?	1	2	3	4	5	6	7
d. How harmful is this event to you?	1	2	3	4	5	6	7

**Table:** 

Someone in charge of you writes a bad report about you	not at all				very much		
a. How likely will this event happen to you in the near future?	1	2	3	4	5	6	7
b. How intentional is this event?	1	2	3	4	5	6	7
c. How controllable is this event by you?	1	2	3	4	5	6	7
d. How harmful is this event to you?	1	2	3	4	5	6	7

**Table:** 

Someone asks you the time	not at all				very much		
a. How likely will this event happen to you in the near future?	1	2	3	4	5	6	7
b. How intentional is this event?	1	2	3	4	5	6	7
c. How controllable is this event by you?	1	2	3	4	5	6	7
d. How harmful is this event to you?	1	2	3	4	5	6	7

**Table:** 

Someone tells you that you are incompetent	not at all				very much		
a. How likely will this event happen to you in the near future?	1	2	3	4	5	6	7
b. How intentional is this event?	1	2	3	4	5	6	7
c. How controllable is this event by you?	1	2	3	4	5	6	7
d. How harmful is this event to you?	1	2	3	4	5	6	7

**Table:** 

You receive a dental appointment in the mail	not at all				very much		
a. How likely will this event happen to you in the near future?	1	2	3	4	5	6	7
b. How intentional is this event?	1	2	3	4	5	6	7
c. How controllable is this event by you?	1	2	3	4	5	6	7
d. How harmful is this event to you?	1	2	3	4	5	6	7

**Table:** 

Someone tells you that you are boring company	not at all				very much		
a. How likely will this event happen to you in the near future?	1	2	3	4	5	6	7
b. How intentional is this event?	1	2	3	4	5	6	7
c. How controllable is this event by you?	1	2	3	4	5	6	7
d. How harmful is this event to you?	1	2	3	4	5	6	7

**Table:** 

Your physical health deteriorates	not at all				very much		
a. How likely will this event happen to you in the near future?	1	2	3	4	5	6	7
b. How intentional is this event?	1	2	3	4	5	6	7
c. How controllable is this event by you?	1	2	3	4	5	6	7
d. How harmful is this event to you?	1	2	3	4	5	6	7

**Table:** 

You find it hard to express yourself with others	not at all				very much		
a. How likely will this event happen to you in the near future?	1	2	3	4	5	6	7
b. How intentional is this event?	1	2	3	4	5	6	7
c. How controllable is this event by you?	1	2	3	4	5	6	7
d. How harmful is this event to you?	1	2	3	4	5	6	7

**Table:** 

You have too many responsibilities to manage	not at all				very much		
a. How likely will this event happen to you in the near future?	1	2	3	4	5	6	7
b. How intentional is this event?	1	2	3	4	5	6	7
c. How controllable is this event by you?	1	2	3	4	5	6	7
d. How harmful is this event to you?	1	2	3	4	5	6	7

**Table:** 

You have an accident	not at all				very much		
a. How likely will this event happen to you in the near future?	1	2	3	4	5	6	7
b. How intentional is this event?	1	2	3	4	5	6	7
c. How controllable is this event by you?	1	2	3	4	5	6	7
d. How harmful is this event to you?	1	2	3	4	5	6	7

**Table:** 

You cannot manage your finances	not at all				very much		
a. How likely will this event happen to you in the near future?	1	2	3	4	5	6	7
b. How intentional is this event?	1	2	3	4	5	6	7
c. How controllable is this event by you?	1	2	3	4	5	6	7
d. How harmful is this event to you?	1	2	3	4	5	6	7

**Table:** 

	Gender	Age	Income

DASS-D	-0.11 (.019)	-0.27 (<.001)	-0.14 (.004)
DASS-A	-0.16 (<.001)	-0.27 (<.001)	-0.10 (.031)
DASS-S	-0.12 (.012)	-0.26 (<.001)	-0.09 (.066)
R-GPTS Persecution	-0.05 (.299)	-0.09 (.072)	0.03 (.490)
RPQ-neutral: likelihood	0.02 (.709)	0.04 (.405)	0.06 (.181)
RPQ-neutral: harm	-0.03 (.578)	-0.10 (.033)	0.03 (.510)
RPQ-neutral: controllability	-0.02 (.632)	0.05 (.262)	0.05 (.253)
RPQ-neutral: intentionality	0.02 (.747)	-0.01 (.816)	0.07 (.169)
RPQ-negative: likelihood	0.00 (.926)	-0.21 (<.001)	-0.04 (.427)
RPQ-negative: harm	-0.15 (.001)	-0.25 (<.001)	0.01 (.910)
RPQ-negative: controllability	-0.03 (.505)	0.03 (.595)	0.01 (.837)
RPQ-negative: intentionality	-0.06 (.196)	-0.16 (<.001)	-0.00 (.937)

Note: Point-biserial correlation for gender (male coded as 1), and Spearman rank correlation for age and income

DASS = Subscales of the Depression Anxiety Stress Scale (D = Depression; A = Anxiety; S = Stress). R-GPTS = Revised Green Paranoid Thoughts Scale. RPQ = Risk Perception Questionnaire.

## Appendix 1 Risk Perception Questionnaire (Chinese translation was used in this study)

Please imagine yourself to be in each scenario and circle the number to indicate your view. There is no right or wrong answer.
